# Disparities in the prevalence and reporting of civilian justifiable firearm homicide

**DOI:** 10.1186/s40621-026-00680-7

**Published:** 2026-04-18

**Authors:** Camerin A. Rencken, Rosanna Smart, Dulani Woods, Terry L. Schell, Andrew R. Morral

**Affiliations:** 1 RAND Corporation, 1776 Main Street, Santa Monica, CA 90401 USA; 2 RAND Corporation, 1200 South Hayes St, Arlington, VA 22202 USA

**Keywords:** Homicide, Defensive gun use, Injury, Firearm violence

## Abstract

**Background:**

Civilian justifiable firearm homicides (CJFH), an important component of defensive gun use, are central to debates on gun policy and ownership but have received limited scholarly attention. This study examines the prevalence and characteristics of CJFH across two national datasets, with a focus on understanding differences across datasets in the relationship of decedent race with the determination of justifiability.

**Methods:**

This cross-sectional study used data from 2016 to 2022 from the National Violent Death Reporting System (NVDRS) and Supplementary Homicide Reports (SHR). Descriptive statistics and logistic regressions assessed the association between coded decedent race and the likelihood that a firearm homicide was coded as justifiable.

**Results:**

After geographically harmonizing the datasets, CJFH incidents were more common in NVDRS (*n* = 1887) than SHR (*n* = 1576). NVDRS had a lower proportion of Black CJFH decedents (58%) compared to SHR (67%). Restricting to a more closely matched set of incidents, adjusted models showed higher odds of coding the decedents of CJFH as Black in SHR (OR: 1.17; 95% CI: 0.93–1.46) than NVDRS (OR: 1.00; 95% CI: 0.82–1.22).

**Conclusions:**

Discrepancies in the racial composition of CJFH across NVDRS and SHR do not arise for other decedent characteristics or among non-CJFH cases. Differences in the coding of CJFH highlight potential issues with evaluating racial disparities and the need to address biases in firearm data infrastructure.

**Supplementary Information:**

The online version contains supplementary material available at 10.1186/s40621-026-00680-7.

## Background

Self-defense is an important motivator for firearm ownership and a central consideration in firearm policymaking. Most gun owners (70%-80%) cite protection as a major reason for firearm ownership [1, 2], with Black and Hispanic gun owners two to three times more likely than their non-Hispanic White counterparts to endorse protection as a very or extremely important reason [3]. Despite its importance, very little is known about defensive gun use (DGU), which can include displaying or firing a firearm to protect oneself, others, or property against a perceived threat or crime [4]. While prior studies have evaluated DGU using surveys [5, 6] or news reports [7], these sources have notable limitations. Survey data reflect only the respondent’s perspective on whether DGU was justified. News reports face biases from selective coverage and incomplete information about incident legitimacy. An issue with both sources is definitional uncertainty, as survey respondents and news outlets do not have standard or uniform criteria for what constitutes legal “defensive gun use,” differing in the extent to which DGU can result from provocation or one’s own criminal activity [8]. Differences in data sources and definitions help explain why DGU prevalence estimates for the U.S. range widely, from 65,000 to more than one million annually [4, 9, 10].

Civilian justifiable firearm homicides (CJFH) coded in the Federal Bureau of Investigation’s (FBI) Supplementary Homicide Reports (SHR) represent one of the only uniformly defined measures of DGU that has been systematically collected across jurisdictions and over time [11]. CJFH are defined as the lawful killing of another person using a firearm in self-defense or in defense of others against someone attempting a forcible felony. To be reported as justifiable, it is not enough to claim self-defense; the self-defense must be in response to a forcible felony, a classification that can vary across states but generally requires use or threat of physical force or violence against a person. While CJFH represent a subset of all DGU, they are officially recorded by law enforcement and thus represent a well-defined and measurable subset with direct implications for self-defense laws and for law enforcement practice.

Evaluations of the effects of gun policy on DGU have also largely relied on the CJFH definition from SHR, with studies using SHR data to evaluate effects of stand-your-ground laws on CJFH overall and by race/ethnicity [12–14]. However, SHR data, while valuable for longitudinal analysis, have been subject to concerns about accuracy and completeness in the context of law enforcement justifiable homicides [15, 16]. Whether SHR’s CJFH data are similarly incomplete has not previously been studied. The lack of research on the quality of CJFH data in the SHR limits our ability to assess the true prevalence and nature of CJFH, which is essential for evaluating the impact of self-defense laws, understanding whether these effects differ across demographic groups, and shaping evidence-based gun policy.

Given the need for reliable data on CJFH to support analysis of DGU and to guide policy and practice, the aims of this paper are twofold. First, we assess the completeness of SHR CJFH records by comparing the prevalence and characteristics of CJFH as measured by the SHR to a more recent alternative administrative dataset: the Center for Disease Control and Prevention’s (CDC) National Violent Death Reporting System (NVDRS). These datasets were chosen as they are, to our knowledge, the only sources for national CJFH data and, in theory, should report such homicides equivalently due to their near parallel coding mechanisms and definitions. Second, we evaluate the consistency of victim race and “justifiability” coding across the datasets by comparing the racial composition of CJFH between the two datasets. Prior research has compared the SHR and NVDRS to improve understanding of law enforcement homicides [17], overall homicides [18], and mass shootings [19], generally finding that the NVDRS has more complete coverage in recent years as well as richer descriptive information on incidents [20]. This study represents the first to compare their reporting of CJFH, with a focus on potential systematic biases in the coding of whether a homicide is “justifiable” depending on decedent race or other characteristics.

## Methods

*Overview.* In this cross-sectional study, we harmonized two administrative datasets temporally and geographically to compare CJFH prevalence and characteristics. We analyzed the composition of CJFH based on similarly measured variables across both datasets, with a focus on decedent race. To assess whether racial differences in CJFH across the datasets were due to differences in how race was measured or differences in geographic coverage, we also examined whether racial differences persisted for other types of firearm homicide or after adjusting for incident characteristics and differences in jurisdictional reporting.

*Data Sources.* Information on firearm homicides came from the SHR and the NVDRS. The NVDRS collects violent death data from death certificates, coroners/medical examiners, and law enforcement. Collection began in 2003 with six participating states, with 50 states reporting by 2020. The SHR, part of the Uniform Crime Reporting system, has collected voluntary homicide data from law enforcement since 1976. Each dataset has significant missingness by jurisdiction, and the pattern of this missingness varies over time. Because our goal was to compare these two data sources, we implemented several data preparation steps to better ensure that differences across datasets were not attributable to differences in the jurisdictions that contribute to each dataset.

For all analyses, we limited to homicides between 2016 and 2022 and excluded homicides where the shooter was an on-duty law enforcement officer. In our “harmonized” dataset, we restricted to homicides from state-years where the state participated in NVDRS (defined as collecting data on 80% or more of violent deaths, a criterion CDC uses for presenting state-level NVDRS statistics), and county-years that had law enforcement agencies participating in SHR (excluding county-years with zero SHR homicides if there were nonzero NVDRS homicides in that county-year). In a second “matched” dataset, we matched the NVDRS and SHR more closely to the subset of firearm homicides that appeared to be captured in both datasets. Specifically, we stratified homicides within the NVDRS and the SHR by the cross-classification of county of occurrence, month and year of injury, victim age in five-year bins, and victim sex. We then restricted our analytic sample to decedents in those strata where the two datasets agreed on the number of firearm homicides within a 10% margin of error. This effectively requires an exact match on number of homicides in each narrow strata except in a few counties that have unusually large numbers of firearm homicides. This matched dataset includes data from state-years where fewer than 80% of violent deaths were included in NVDRS, so long as the homicides were recorded in both datasets. See Table 1 for information on how sample restrictions affected the number of homicides used in the analysis.

*Exposure (Decedent Coded Race) and Outcome (Homicide Coded as Justifiable).* Among firearm homicides, we looked at the relationship between the coding of the decedent race and whether the firearm homicide was coded as justifiable. We focused on victim race rather than victim and offender race due to a high degree of missingness on offender variables that is directly related to whether an offender is known and hence the justifiability determination (see Additional File 1).

Coded decedent race was categorized as Black or non-Black; further granularity was not possible due to poor alignment in race coding across datasets. We did not code for ethnicity given the high levels of missingness on victim ethnicity in SHR (28.4% missing/unknown). We treated decedents in the NVDRS who were coded as two or more races as non-Black (SHR does not have a multiple-race category), but results were not sensitive to alternative treatments. Information on whether an incident was coded as justifiable came from yes/no variables in each dataset specific to this question. Coding manuals for NVDRS and SHR use the same definition of justifiable homicide, and NVDRS coders are further instructed to code homicides as justifiable only if the SHR or other law enforcement reports explicitly categorize the homicide as justifiable [21].

*Statistical Analysis.* Descriptive statistics are reported as medians with interquartile ranges or as frequencies and percentages. We used logistic regressions to estimate the association between coded decedent race and the classification of a homicide as justifiable.

**Table 1:** Flowchart of Study Population Selection

	NVDRS (2016–2022)		SHR (2016–2022)^a^	
	Total (*n*)	CJFH (%)	Total (*n*)	CJFH (%)
**Firearm homicides**	93,517	2.3%	89,042	2.6%
Drop state-years with < 80% reporting to NVDRS^b^	80,642	2.5%	64,069	2.5%
Drop county-years with no SHR reporting	73,266	2.6%	64,069	2.5%
Drop law enforcement involved homicides	69,450	2.7%	62,266	2.5%
**Harmonized analytic dataset**	69,450	2.7%	62,266	2.5%
**Matched dataset**	55,937	2.2%	55,311	2.6%

CJFH = civilian justifiable firearm homicides. NVDRS = National Violent Death Reporting System. SHR = Supplementary Homicide Reports. The CJFH (%) column indicates the percentage of total homicide incidents that are coded as CJFH within each dataset and sample restriction

^a^ SHR converted from incident- to victim-level

^b^ We apply the 80% reporting threshold as this is the common criteria applied for presentation of state-level statistics by CDC; three states collected data on 80% or more of violent deaths as was required for funding over the following years prior to full participation: Illinois (2016–2019), Pennsylvania (2016–2019), and Washington (2016–2017)

We estimated four regression specifications. First, we examined unadjusted associations using the harmonized dataset. Second, to better assess whether differences across datasets were specific to the association between victim race and justifiable classification, we adjusted for additional incident characteristics, including year of occurrence, victim age, sex, and relationship to the suspect. Last, to better ensure that any differences across data sources in rates of justifiable homicides by race can be interpreted as differences in how they were coded rather than differences in the underlying incidents captured by each dataset, we estimated the same unadjusted and adjusted models within the matched dataset restricted to those homicides that were likely captured in both SHR and NVDRS. We clustered standard errors at the county level and completed analyses using R version 4.4.1 and Stata 19 software [22].

*Ethical Review.* This study was determined exempt (Category 4) by RAND’s Institutional Review Board.

## Results

Examining both full datasets from 2016 to 2022, the NVDRS recorded 93,517 firearm homicides, compared to 89,042 reported by the SHR (Table 1). For comparison, the National Vital Statistics System, considered a near-census of deaths in the United States, recorded 117,322 firearm homicides over that period.

Based on our harmonized dataset—limited to the 290 state-years with sufficient coverage in both sources—the NVDRS recorded 69,450 firearm homicides between 2016 and 2022, compared to 62,266 reported by the SHR. Of these, 1,887 homicides (2.7%) in the NVDRS and 1,576 homicides (2.5%) in the SHR were classified as CJFH (Table 1). Maine had the highest CJFH percentage in NVDRS (10.1% vs. 5.3% in SHR), while Nevada had the highest in SHR (8.4% vs. 8.6% in NVDRS). In both datasets, 2022 was the year with the greatest proportion of firearm homicides classed as CJFH (3.0% in the NVDRS and 2.8% in the SHR).

*Descriptive Results.* NVDRS coded 58.4% (1,102) of CJFH decedents as Black vs. 66.6% (1,049) in SHR, an 8-percentage-point difference. No such difference was seen in non-CJFH homicides (both approximately 67% Black, Table 2). Among other decedent demographics, CJFH and non-CJFH cases across NVDRS and SHR were more consistent: the median age was 31 years in NVDRS versus 30 years in SHR, and between 96% and 97% of the decedents were male in both datasets. In 26.9% of NVDRS CJFH cases, the decedent and offender were reported to be strangers, compared to 31.9% in SHR (Table 2); although coding of this variable may differ across these datasets and comparisons should be interpreted cautiously [18]. In the NVDRS, 96.0% of CJFH cases involved a single victim, compared to 92.4% of non-CJFH; in the SHR, the percentages were 94.5% and 86.6%, respectively.

**Table 2:** Descriptive Characteristics of Harmonized Dataset Stratified by Data Source and Civilian Justifiable Firearm Homicide (2016–2022)

Data Source	Civilian Justifiable Firearm Homicide		Civilian Non-Justifiable Firearm Homicide	
	NVDRS (*n* = 1,887)	SHR (*n* = 1,576)	NVDRS (*n* = 67,563)	SHR (*n* = 60,690)
**Decedent: Black** (n, col %)	1,102 (58.4%)	1,049 (66.6%)	45,241 (67.0%)	40,496 (66.7%)
**Decedent: Non-Black** (n, col %)	756 (40.1%)	518 (32.9%)	20,642 (30.6%)	19,281 (31.8%)
**Decedent: Male** (n, col %)	1,828 (96.9%)	1,516 (96.2%)	56,466 (83.6%)	50,277 (82.8%)
**Decedent: Female** (n, col %)	59 (3.1%)	59 (3.7%)	11,097 (16.4%)	10,326 (17.0%)
**Decedent: Age**,** years** (median, IQR)	31.0 (23.0–40.0)	30.0 (23.0–39.0)	29.0 (22.0–39.0)	29.0 (22.0–39.0)
**Offender: Stranger** (n, col %)	507 (26.9%)	503 (31.9%)	3,280 (4.9%)	4,233 (7.0%)
**Offender: Non-stranger ** (n, col %)	833 (44.1%)	619 (39.3%)	19,020 (28.1%)	18,144 (29.9%)

IQR = the interquartile range. NVDRS = National Violent Death Reporting System. SHR = Supplementary Homicide Reports. Process for dataset harmonization is described in the text and Table 1. Race, sex, and victim-offender relationships do not sum to 100% due to incidents with unknown or missing values on these measures

*Modeling Results.* In initial unadjusted models (Table 3), Black firearm homicide decedents had lower odds of being classified as justifiable homicide victims compared with non-Black decedents in the NVDRS (odds ratio [OR]: 0.67; 95% confidence interval [CI]: 0.55–0.80). In SHR, Black decedents had 0.96 times the odds (95%-CI: 0.77–1.21). After adjusting for incident characteristics, Black decedents had 0.92 times the odds (95%-CI: 0.79–1.07) of being classified as justifiable firearm homicide victims in NVDRS, and 1.18 times the odds (95%-CI: 0.96–1.46) in SHR compared to non-Black decedents. Across all models, the SHR consistently showed a larger odds ratio between Black decedent race and likelihood of the death being coded as justifiable compared to the NVDRS. This disparity across datasets did not exist for other demographics. For example, the association between age and CJFH coding was consistent in both the NVDRS and the SHR (see Additional File 2).

**Table 3:** Odds Ratios for Justifiable Firearm Homicide Classification by Victim Race (Black vs. non-Black): Harmonized NVDRS and SHR (2016–2022)

	NVDRS	SHR
	Odds Ratio (95% CI)	Odds Ratio (95% CI)
Harmonized dataset, unadjusted	0.67	0.96
	(0.55–0.80)	(0.77–1.21)
Harmonized dataset, adjusted for incident characteristics^a^	0.92	1.18
	(0.79–1.07)	(0.96–1.46)
Matched dataset, unadjusted^b^	0.74	0.97
	(0.58–0.95)	(0.76–1.24)
Matched dataset, adjusted^a, b^	1.00	1.17
	(0.82–1.22)	(0.93–1.46)

CI = confidence interval. NVDRS = National Violent Death Reporting System. SHR = Supplementary Homicide Reports. Process for dataset harmonization is described in the text and Table 1. Results reported as average marginal effects are included in Additional File 4. Standard errors are clustered at the county level

^a^Adjusted analyses control for offender-victim relationship, age, victim sex, and year fixed effects. All model coefficients from the regression are reported in Additional File 2

^b^Data in the matched dataset were restricted to matched strata by county, time, age, and sex where NVDRS and SHR firearm homicide counts closely aligned

To better ensure these observed differences in the relationship of coding decedent race and coding a firearm homicide as justifiable, we replicated these analyses on the matched subset of cases pulled from strata (county, time, demographic group) where the two datasets recorded nearly the same number of firearm homicides. In this dataset we have high confidence that both datasets captured the same incidents, and thus differences likely reflect variation in how the same incidents are being coded. In these more closely matched datasets, approximately 2.5% of all firearm homicides were classified as CJFH, consistent with the proportion observed in the primary analyses (Table 1). Black decedents had 0.74 times the odds (95%-CI: 0.58–0.84) of being classified as justifiable firearm homicide victims in NVDRS, and 0.97 times the odds (95%-CI: 0.76–1.24) in SHR compared to non-Black decedents (Table 3). Adjusted models showed similar differences: the association of Black victim race with justifiable determination in the NVDRS was zero and not statistically significant (95% CI: 0.82–1.22), while Black victim race was associated with a large (albeit not statistically significant) increased likelihood of classifying a firearm homicide as justifiable in SHR (OR: 1.17, 95%-CI: 0.93–1.46). Thus, even when looking at (plausibly) the same set of homicides, the SHR is more likely than the NVDRS to classify Black decedents as justifiably killed. These findings hold under alternative ways of handling victims with multiple races indicated in the NVDRS (see Additional File 3).

## Discussion

Permit-less concealed carry and stand-your-ground laws have been widely adopted, partly to facilitate DGU [23, 24]. This study provides a critical comparison of the NVDRS and SHR datasets to examine their utility for supporting analyses of such policy effects and to investigate patterns in CJFH. The findings highlight key substantive differences between the datasets, particularly in the racial composition of CJFH decedents (but not other firearm homicide victims). The fact that two different data sources covering essentially the same firearm homicides generate substantially different estimates about the extent to which Black decedents are killed justifiably or not is a concern for both policy and research, regardless of whether these estimates are statistically significantly different from an odds ratio of one in either data source.

Between 2016 and 2022, the NVDRS consistently reported more CJFH incidents than the SHR (1,887 vs. 1,576, respectively). This finding parallels results from prior research showing the NVDRS captures more law enforcement-related homicides than the SHR [17], likely due to incomplete coverage of law enforcement agencies in the SHR. When restricting to matched datasets—retaining only strata with similar case counts by county, month and year of occurrence, and victim age, and therefore likely more closely reflecting reporting agencies with complete homicide data—the SHR reports slightly more CJFH cases than the NVDRS (1,412 vs. 1,245, respectively) but fewer firearm homicides overall.

Multiple factors could contribute to differences in CJFH prevalence between the datasets. For instance, the SHR may differentially classify certain homicides due to differences in legal definitions or coding practices, especially in states with “excusable homicide” laws that diverge from the FBI’s definitions [25]. In addition, the SHR is completed shortly after the homicide occurs, often before crucial details, such as a court ruling, are available, a limitation not shared by NVDRS [18]. The NVDRS also benefits from more data sources (e.g., coroner and medical examiner reports) which may provide a more complete picture of incidents. These differences highlight the need for standardized reporting practices across all states and jurisdictions to ensure that data on justifiable homicides are accurate and comparable.

We observed notable differences in the racial composition of CJFH victims across the two datasets, differences that did not arise for other demographic characteristics. Although the number of Black CJFH decedents was very similar in the SHR and NVDRS (1049 and 1102, respectively), the number of non-Black CJFH decedents differed considerably (518 versus 756). In the SHR, after adjusting for incident characteristics, Black victims were more likely to have their deaths classified as justifiable—a pattern not seen in the NVDRS. Even when restricting to those cases that were most likely to have been captured in both datasets, Black decedents remained substantially more likely to have their deaths coded as justifiable in the SHR than in the NVDRS.

The reason for this discrepancy is unclear, but it is concerning in the broader context of racial disparities within the criminal justice system [26, 27]. Although SYG laws are codified in statutory frameworks, their interpretation and application vary by jurisdiction and likely hinge on subjective judgments about reasonableness that could be affected by racial biases. For instance, Yakubovich et al. found that self-defense claims under SYG laws were less likely to be upheld when the victim was White, a disparity exacerbated when the claimant was a racial minority [24]. Other analyses of SHR data from have found that homicides involving White offenders and Black victims were significantly more likely to be ruled justifiable compared to other racial combinations [14]. While previous research has shown that Black decedents are more likely to be classified as justifiably killed in the SHR, our study is the first to demonstrate that this pattern does not hold as strongly in a different dataset—one that is intended to use the same definition but involves different coders and additional contextual information in some cases.

The differences in coding across data sources may reflect systematic variations in perceptions or reporting practices among police agencies versus medical examiner or coroner offices. For example, compared to the NVDRS coders, police reports may more frequently link decedents’ race and criminal behavior. While we do not know which set of codes is more accurate, the SHR data is more consistent with societal racial stereotypes and thus could reflect some type of cognitive bias. If so, these biases may have implications for racial equity because the police determination that a homicide was justified, i.e., that the decedent was engaged in a crime, largely determines whether anyone will be punished for the homicide.

It is important to acknowledge that we cannot definitively determine which dataset most accurately reflects the true circumstances of each case. While the SHR has more comprehensive historical data that is necessary for understanding long-term national trends [20], our results indicate that, since 2016, the NVDRS captures a larger set of firearm homicides than SHR. The NVDRS also has advantages in integrating information from multiple sources, incorporating updates when new information becomes available. In contrast, the SHR reflects data submitted voluntarily and directly by law enforcement agencies, which may be more difficult to revise as information about case circumstances updates. Differences in data availability, coding practices, and the extent to which SHR reflects the initial determination made by law enforcement and NVDRS the final incident determination may all contribute to the observed discrepancies and racial disparities between the two sources. Future research that takes a more detailed look at the characteristics, medical examiner notes, and law enforcement narratives for cases coded as CJFH in NVDRS but not SHR may help clarify which candidate explanations are more plausible.

Another notable finding is that CJFH incidents often involve individuals known to each other. In both datasets over half of CJFH involved non-strangers or unknown relationships. Since both datasets typically report only one suspect-victim relationship, this pattern may be understated. Policies based on the assumption that CJFH primarily involves strangers risk overlooking the role of interpersonal dynamics. Targeted interventions may be needed in this context, such as public awareness campaigns that address the risks of firearms in domestic disputes.

This study has several limitations. First, both datasets suffer from some level of systematic coverage error and do not include all incidents of CJFH. While this limitation may affect our epidemiological findings, these are currently the most comprehensive and authoritative datasets available for studying CJFH. Second, we were unable to link SHR and NVDRS records at the individual case level, thus our conclusions are focused on aggregate comparisons between the datasets rather than on individual cases. However, we did compare closely matched cases that shared multiple characteristics (county of death occurrence, year and month of injury, victim sex, and victim age) in sensitivity analyses. Third, while CJFH represents one form of defensive gun use for which we have data, there are other aspects, such as the number of crimes prevented, that we have not included but that warrant investigation when considering the use of firearms for self-defense. These issues underscore the need for enhanced data consistency and quality address the risks of firearms in domestic settings.

## Conclusions

This study compares two datasets—the Supplementary Homicide Reports (SHR) and the National Violent Death Reporting System (NVDRS)—to assess how civilian firearm justifiable homicides (CJFH) are coded. Notable differences in the racial distribution of CJFH victims were observed between the two datasets, while other demographic characteristics remained consistent. For researchers interested in justifiable homicide, each dataset offers distinct advantages and limitations. The NVDRS includes more detailed information on incident characteristics, enhancing its value for understanding the context of CJFH. The SHR offers a longer historical record, beginning in 1976, supporting analyses of longer-term trends. These findings highlight key considerations for researchers and practitioners using these data sources to monitor trends and inform public health approaches to firearm-related violence and defensive gun use.

## Supplementary Information

Supplementary Material 1

Supplementary Material 2

Supplementary Material 3

Supplementary Material 4

## Data Availability

The Supplementary Homicide Reports are publicly available at Kaplan, Jacob, 2025, “Summary Reporting System (SRS) - Supplementary Homicide Reports (SHR)”, https://doi.org/10.7910/DVN/YB76AT, Harvard Dataverse, V1. The underlying National Violent Death Reporting System data used in this study’s analyses are not publicly available because they require a restricted-use agreement; however, the data are made available for research purposes through an application for access to the Centers for Disease Control and Prevention.

## Abbreviations

CDC: Center for Disease Control and Prevention
CI: Confidence interval
CJFH: Civilian justifiable firearm homicides
DGU: Defensive gun use
FBI: Federal Bureau of Investigation
NVDRS: National Violent Death Reporting System
SHR: Supplementary Homicide Reports
SYG: Stand your ground
