# Supplementary material for: Disparities in the prevalence and reporting of civilian justifiable firearm homicide
Source: Inj Epidemiol. 2026 Apr 18;13:44. doi: 10.1186/s40621-026-00680-7 (PMC13217811; doi:10.1186/s40621-026-00680-7)
Supplement: Supplementary file 1 — Supplementary Material 1 [file 40621_2026_680_MOESM1_ESM.docx]

**Additional File 1. Prevalence of Victim-Offender Racial Dyads in Justifiable and Non-Justifiable Civilian Firearm Homicides, by Dataset**

|  | **Civilian Justifiable Firearm Homicide** | | **Civilian Non-Justifiable  Firearm Homicide** | |
| --- | --- | --- | --- | --- |
| **Offender-Victim Race**  n (col %) | **NVDRS** | **SHR** | **NVDRS** | **SHR** |
| **Panel A: Harmonized Datasets** | | | | |
| NonBlack-NonBlack | 464 (24.6%) | 437 (27.7%) | 8,324 (12.3%) | 20,303 (15.4%) |
| Black-Black | 637 (33.8%) | 832 (52.8%) | 16,847 (24.9%) | 37,940 (28.8%) |
| NonBlack-Black | 146 (7.7%) | 169 (10.7%) | 1,063 (1.6%) | 3,002 (2.3%) |
| Black-NonBlack | 79 (4.2%) | 70 (4.4%) | 3,093 (4.6%) | 6,878 (5.2%) |
| Missing-NonBlack | 213 (11.3%) | 11 (0.7%) | 9,225 (13.7%) | 14,016 (10.6%) |
| Missing-Black | 319 (16.9%) | 48 (3.0%) | 27,331 (40.5%) | 46,946 (35.6%) |
| NonBlack-Missing | 10 (0.5%) | 7 (0.4%) | 241 (0.4%) | 532 (0.4%) |
| Black-Missing | 8 (0.4%) | 2 (0.1%) | 270 (0.4%) | 516 (0.4%) |
| Missing-Missing | 11 (0.6%) | 0 (0.0%) | 1,169 (1.7%) | 1,583 (1.2%) |
| **Panel B: Matched Datasets** | | | | |
| NonBlack-NonBlack | 283 (22.7%) | 404 (28.6%) | 6,138 (11.2%) | 9,584 (17.8%) |
| Black-Black | 430 (34.5%) | 722 (51.1%) | 13,079 (23.9%) | 16,911 (31.4%) |
| NonBlack-Black | 98 (7.9%) | 163 (11.5%) | 806 (1.5%) | 1,482 (2.7%) |
| Black-NonBlack | 53 (4.3%) | 64 (4.5%) | 2,403 (4.4%) | 3,165 (5.9%) |
| Missing-NonBlack | 157 (12.6%) | 11 (0.8%) | 9,210 (16.8%) | 5,049 (9.4%) |
| Missing-Black | 209 (16.8%) | 43 (3.0%) | 21,831 (39.9%) | 17,114 (31.8%) |
| NonBlack-Missing | 5 (0.4%) | 3 (0.2%) | 177 (0.3%) | 178 (0.3%) |
| Black-Missing | 3 (0.2%) | 2 (0.1%) | 196 (0.4%) | 155 (0.3%) |
| Missing-Missing | 7 (0.6%) | 0 (0.0%) | 852 (1.6%) | 261 (0.5%) |

NOTES: NVDRS = National Violent Death Reporting System. SHR = Supplementary Homicide Report. Process for dataset harmonization is described in the text and Table 1. Processes for constructing the harmonized datasets (Panel A) and group-matched datasets (Panel B) are described in the text.
