# Supplementary material for: Disparities in the prevalence and reporting of civilian justifiable firearm homicide
Source: Inj Epidemiol. 2026 Apr 18;13:44. doi: 10.1186/s40621-026-00680-7 (PMC13217811; doi:10.1186/s40621-026-00680-7)
Supplement: Supplementary file 2 — Supplementary Material 2 [file 40621_2026_680_MOESM2_ESM.docx]

**Additional File 2. Association between Decedent Race and Justifiable Firearm Homicide Coding in NVDRS and SHR, Adjusted Model Results for All Coefficients**

|  | **NVDRS** | | **SHR** | |
| --- | --- | --- | --- | --- |
|  | **Odds Ratio** | **95% CI** | **Odds Ratio** | **95% CI** |
| Black Decedent | 0.92 | 0.79–1.07 | 1.18 | 1.96–1.46 |
| Offender – Victim Relationship  Non-stranger  Stranger | 4.52  13.74 | 3.69–5.54  11.10–17.01 | 3.45  10.34 | 2.70–4.40  7.79–13.72 |
| Victim Female | 0.12 | 0.09–0.15 | 0.17 | 0.13–0.23 |
| Victim Age | 1.00 | 1.00–1.00 | 1.00 | 1.00–1.00 |
| Incident Year |  |  |  |  |
| 2017 | 1.04 | 0.80–1.35 | 1.03 | 0.78–1.36 |
| 2018 | 1.11 | 0.84–1.47 | 1.26 | 0.93–1.69 |
| 2019 | 1.19 | 0.96–1.49 | 1.22 | 0.89–1.67 |
| 2020 | 1.34 | 1.06–1.69 | 1.10 | 0.82–1.48 |
| 2021 | 1.41 | 1.11–1.78 | 1.12 | 0.80–1.59 |
| 2022 | 1.49 | 1.17–1.90 | 1.31 | 1.00–1.70 |

NOTES: CI = confidence interval. NVDRS = National Violent Death Reporting System. SHR = Supplementary Homicide Report. Process for dataset harmonization is described in the text and Table 1.
