# Supplementary material for: Disparities in the prevalence and reporting of civilian justifiable firearm homicide
Source: Inj Epidemiol. 2026 Apr 18;13:44. doi: 10.1186/s40621-026-00680-7 (PMC13217811; doi:10.1186/s40621-026-00680-7)
Supplement: Supplementary file 3 — Supplementary Material 3 [file 40621_2026_680_MOESM3_ESM.docx]

**Additional File 3.** **Odds Ratios for Justifiable Firearm Homicide Classification by Victim Race (Black vs non‑Black), Alternative Methods for Handling Decedents with Multiple Races Indicated in NVDRS**

|  |  |  |  |
| --- | --- | --- | --- |
|  | **Primary Analysis** | **Alternative Using Multiple Race Label** | **Alternative Controlling for Multiple Race** |
|  | **(1)** | **(2)** | **(3)** |
| Harmonized dataset, unadjusted | 0.67 | 0.67 | 0.67 |
|  | (0.55–0.80) | (0.56–0.81) | (0.56–0.81) |
|  |  |  |  |
| Harmonized dataset, adjusted^a^ | 0.92 | 0.93 | 0.93 |
|  | (0.79–1.07) | (0.80–1.09) | (0.77–1.08) |
|  |  |  |  |
| Matched subset of incidents, unadjusted^b^ | 0.74 | 0.76 | 0.75 |
|  | (0.58–0.95) | (0.59–0.97) | (0.59–0.96) |
|  |  |  |  |
| Matched subset of incidents, adjusted^a,b^ | 1.00 | 1.01 | 1.01 |
|  | (0.82–1.22) | (0.84–1.123) | (0.83–1.23) |
|  |  |  |  |

NOTES: CI = confidence interval. NVDRS = National Violent Death Reporting System. Process for dataset harmonization is described in the text and Table 1. Column (1) replicates the primary analysis results from Table 3. Column (2) assigns as Black those decedents who had a multiple race label that included “African American” among the multiple races listed. Column (3) includes an additional covariate that is a dummy variable for whether multiple race was indicated.

For the harmonized dataset, the proportions of victims classified as Black using multiple race labels are 59.5% (1,123 of 1,887) for justifiable firearm homicides and 67.7% (45,729 of 67,563) for non-justifiable firearm homicides; respective percentages for the primary analysis were 58.4% and 67.0%. For the matched dataset, the proportions of victims classified as Black using multiple race labels are 60.5% (753 of 1,245) for justifiable firearm homicides and 66.1% (36,157 of 54,692) for non-justifiable firearm homicides; respective percentages for the primary analysis were 59.9% and 66.8%.

^a^Adjusted analyses control for offender-victim relationship, age, victim sex, and year fixed effects.

^b^Data in the matched subset were restricted to matched strata by county, time, age, and sex where NVDRS and SHR firearm homicide counts closely aligned.
