# Supplementary material for: Disparities in the prevalence and reporting of civilian justifiable firearm homicide
Source: Inj Epidemiol. 2026 Apr 18;13:44. doi: 10.1186/s40621-026-00680-7 (PMC13217811; doi:10.1186/s40621-026-00680-7)
Supplement: Supplementary file 4 — Supplementary Material 4 [file 40621_2026_680_MOESM4_ESM.docx]

**Additional File 4.** **Average Marginal Effects for Justifiable Firearm Homicide Classification by Victim Race (Black vs non‑Black), Expressed as Percentage Point Differences**

|  | **NVDRS** | **SHR** |
| --- | --- | --- |
|  | **Average marginal effect  (95% CI)** | **Average marginal effect**  **(95% CI)** |
| Harmonized dataset, unadjusted | -1.09 | -0.09 |
|  | (-1.49, -0.68) | (-0.65, 0.47) |
|  |  |  |
| Harmonized dataset, adjusted for incident characteristics^a^ | -0.21 | 0.41 |
|  | (-0.60, 0.17) | (-0.14, 0.95) |
|  |  |  |
| Matched subset of incidents, unadjusted^b^ | -0.65 | -0.07 |
|  | (-1.17, -0.14) | (-0.57, 0.53) |
|  |  |  |
| Matched subset of incidents, adjusted^a,b^ | -0.01 | 0.37 |
|  | (-0.43, 0.42) | (-0.20, 0.95) |
|  |  |  |

NOTES: CI = confidence interval. NVDRS = National Violent Death Reporting System. Process for dataset harmonization is described in the text and Table 1. This table replicates the analyses used to produce Table 3 in the main text but reports model results as average marginal effects, specifically the difference in percent of firearm homicides that are ruled CJFH for Black decedents minus the percent for non-Black decedents.

^a^Adjusted analyses control for offender-victim relationship, age, victim sex, and year fixed effects, and average marginal effects for decedent race are calculated using the observed value of the covariates.

^b^Data in the matched subset were restricted to matched strata by county, time, age, and sex where NVDRS and SHR firearm homicide counts closely aligned.
